# Characterization of Lactic Acid Bacteria Isolated From the Gastrointestinal Tract of a Wild Boar as Potential Probiotics

**DOI:** 10.3389/fvets.2020.00049

**Published:** 2020-02-11

**Authors:** Miao Li, Yi Wang, Hongyu Cui, Yongfeng Li, Yuan Sun, Hua-Ji Qiu

**Affiliations:** State Key Laboratory of Veterinary Biotechnology, Harbin Veterinary Research Institute, Chinese Academy of Agricultural Sciences, Harbin, China

**Keywords:** lactic acid bacteria, probiotic, adhesion ability, antimicrobial activity, wild boar intestine

## Abstract

Lactic acid bacteria (LAB) are major microorganisms used for probiotic purposes and prime parts of the human and mammalian gut microbiota, which exert important health-promoting effects on the host. The present study aimed to evaluate and compare the probiotic potential and safety of LAB strains isolated from the gastrointestinal tract of a wild boar from the Greater Khingan Mountains, China. Amongst all of the isolated LAB strains, five isolates identified as *Lactobacillus mucosae, Lactobacillus salivarius, Enterococcus hirae, Enterococcus durans*, and *Enterococcus faecium*, were remarkably resistant to acid and bile salt. The probiotic characteristics (including adhesion capability, antimicrobial activities, autoaggregation, and coaggregation abilities), and safety properties (including hemolytic activity, antibiotic resistance, absence/presence of virulence factors, and *in vivo* safety) were evaluated. The results showed that all five isolates exhibited high adhesive potential, remarkable aggregation capacity, and antibacterial activities. Upon assessment of the safety, these strains were negative for hemolytic activity and all tested virulence genes. *In vivo* safety assessment showed no adverse effects of isolated strains supplementation on the body weight gain and organ indices of the treated mice. This study revealed that these LAB isolates, especially *L. salivarius* M2-71, possess desirable probiotic properties and have great potentials for the development of feed additives for animals to promote health.

## Introduction

In the past decades, antibiotics abuse in domestic animal feeding has been a major threat to animal health and welfare as well as the environment (1). Since probiotics have positive effects on preventing certain diseases and maintaining good health, they have become one of the widely applied feed additives as an alternative strategy to antibiotics. The positive roles of probiotics for humans and animals, especially with the development of commercial-scale animal husbandry, have widely been accepted. Many studies have shown that the supplementation of probiotic products can improve feed efficiency and growth rate and reduce intestinal infections in many various animals (2–5).

Lactic acid bacteria (LAB) comprise a great variety of genera, and they are widely used as probiotics with a long history (6). LAB exist in a wide range of habitats, including gastrointestinal (GI) tracts, oral cavities, vaginal tracts of humans and animals, fermented foods, silages, and composts (7). They possess various health benefits to the host, such as enhancement of immune function (8), improved digestion (9), management of inflammatory bowel diseases (10), alleviation of constipation (11), and strengthening the mucosal barrier (12). Some LAB isolates even hold anticancer or antidiabetic effects (13, 14). Therefore, LAB have been taken as the best candidate probiotics.

LAB are characterized by producing lactic acid and metabolites, including antioxidants, organic acids, and antimicrobial compounds, modulating and improving intestinal microbial balance (15). LAB feed supplementation improves meat production through growth rate promotion, increased feed conversion, and disease prevention (2). For example, probiotics have been used in pig feed for improving the immune status by reducing harmful microbes in their intestines (16). Moreover, the administration of probiotics to dairy calves results in improvement daily weight gain and resolution of diarrhea (17). Similar results have also been reported in other animals, including poultry (2, 18), ostriches (19), raccoon dogs (20), and silver foxes (20). LAB have widely been applied in animal feed. At present, screening for new probiotics, especially from some undeveloped species has become an ongoing practice (21).

The Greater Khingan Mountains are located in the northernmost prefecture administrative region in China. The area possesses the largest forest, which has rich species, diversiform ecosystems, and sparse population. It is expected to isolate probiotic strains with natural and excellent characteristics. Wild boars could make full use of natural resources in forest areas and have a strong adaptability to their ecological environment conditions. However, there are few studies on probiotics from wild boar. Therefore, the purpose of the current work is to identify the LAB strains isolated from the wild boar in the Greater Khingan Mountains and evaluate their probiotic potentials and safety.

## Materials and Methods

### Bacterial Strains, Cells, and Culture Conditions

The culture medium for isolation of LAB is de Man, Rogosa and Sharpe (MRS) broth. All pathogenic bacteria were purchased from the BeNa Culture Collection (BNCC, China) and cultured according to the instructions. The pathogens including *Staphylococcus aureus* (ATCC 6538P, ATCC 25923), enteropathogenic *Escherichia coli* (BNCC 337304, ATCC 8379), and *Salmonella typhimurium* (ATCC 14028, ATCC 19585) were used for the evaluation of coaggregation and bacteriostatic activities. These pathogenic strains, indicator bacteria used in the antimicrobial assays, were incubated in brain heart infusion (BHI) medium at 37°C. As a reference strain, *Lactobacillus acidophilus* (ATCC 4356) which is able to colonize the intestine of human was included in the study for comparison (22, 23). Caco-2 cells were cultured in Minimum Essential Medium (MEM) (Gibco, USA), 20% of fetal bovine serum (FBS) (Gibco, USA) and 10 mM HEPES (Sigma-Aldrich, Germany). IPEC-J2 (a porcine intestinal enterocytes cell line) cells were grown in Dulbecco's Modified Eagle Medium (DMEM) (Gibco, USA) with 10% FBS. The Caco-2 and IPEC-J2 cell lines were incubated at 37°C with 5% CO_2_.

### Animals

The GI tract samples including the duodenum, ileum, cecum, and colon were obtained from a healthy wild boar of the Greater Khingan Mountains immediately after slaughter in a commercial slaughterhouse. The serum sample of the wild boar was confirmed to be negative for common porcine viruses, including African swine fever virus (ASFV), porcine reproductive and respiratory syndrome virus (PRRSV), classical swine fever virus (CSFV), pseudorabies virus (PRV), and porcine circovirus type 2 (PCV2) by PCR. For safety evaluation of LAB, 6–7-week-old BALB/c mice (purchased from Liaoning Changsheng Biotechnology Co., Ltd.) were randomly divided into six groups (5 mice per group). The mice were kept in an environmentally controlled room maintained in a cycle of 12 h of light and 12 h of dark. The approval for animal experiments was obtained from the Experimental Animal Ethics Committee of Harbin Veterinary Research Institute (HVRI), Chinese Academy of Agricultural Sciences with the license SYXK (Heilongjiang) 2011022.

### Bacterial Isolation From Wild Boar

All the GI tract samples were transported directly to the laboratory for microbial analysis at 4°C. The LAB were isolated and grown overnight in MRS medium and spotted onto MRS agar plates. All the isolated bacteria were incubated at 37°C for 24–48 h.

### Molecular Identification of LAB

The LAB were grown overnight and the genomic DNA was extracted from 1.5 ml cultures using a bacterial genome extraction kit (Tiangen, China) following the manufacturer's protocols. The identification of isolates was analyzed based on the 16S rDNA gene amplification with primer pair: 16S rDNA-F (5′-AGA GTT TGA TCC ATG GCT CAG-3′)/16S rDNA-R (5′-AAG GAG GTG ATC CAG CC-3′). Then PCR was performed using a previously described method (24). The sequences for the amplified 16S rDNA were searched using Blast in the NCBI databases to compare with the registered sequences.

### Acid and Bile Salt Resistance Assays

Acid survivability of the identified species was assessed on the basis of the method described by Dowarah et al. (25). To evaluate the tolerance capacity under acidic pH condition, the acidic pH of MRS broth was adjusted to pH 3.0 and 6.5 using 1 M HCl. One hundred μl of overnight grown LAB was added into 5 ml MRS medium with different pH and incubated for 4 h at 37°C. Optical density at 600 nm (OD_600nm_) was measured for monitoring the growth kinetics. The above tests were carried out in triplicate for each strain.

The survivability of these isolates in bile salt was measured according to Nami et al. (26). Briefly, the MRS medium was prepared with 0.3% and without bile salt. Two media inoculated with 1% of culture were incubated for 4 h at 37°C. OD_600nm_ was measured using a BioSpectrometer (Eppendorf, Germany). The growth rate was calculated as follow: % of growth = Growth in bile salt medium/Growth in control medium × 100.

### Autoaggregation and Coaggregation Assays

Autoaggregation properties were determined following the method described by Collado et al. (27), with some modifications. In short, the isolates were cultured in MRS medium at 37°C for 18 h. The cultures were harvested by centrifugation at 5,000 × *g* for 15 min, washed 3 times using phosphate-buffered saline (PBS), and resuspended in 2 ml PBS to an OD_600nm_ of 0.25 ± 0.05. Then the bacterial solution was incubated at room temperature (RT) and was measured at different time points (0, 2, 4, 6, 10, and 24 h). At each time point, 100 μl of the upper part of the bacterial suspension was transferred to a disposable cuvette and the absorbance (A) was measured at 600 nm. The autoaggregation rates were determined as follows: [(A_X_ – A_y_)/A_x_] × 100 (where A_x_ denotes the absorbance at time (*t*) = 0, A_y_ denotes the absorbance at *t* = 2, 4, 6, 10, or 24 h).

The bacterial suspensions for coaggregation were prepared in the same way as above. Then, 2 ml suspension of different isolated strains and the three pathogenic strains (*Staphylococcus aureus* ATCC 6538P, enteropathogenic *Escherichia coli* BNCC 337304, and *Salmonella typhimurium* ATCC 14028) were mixed and incubated at RT. At different time points (0, 2, 4, 6, 10, and 24 h), the absorbance of the mixture was monitored and determined. The coaggregation (%) was calculated as follows: [(Apro + Apat)-Amix]/(Apro + Apat) × 100, where Apro + Apat represents the absorbance of the mixture of the LAB and the pathogen at time 0 h, and Amix denotes the absorbance of the LAB mixture and the pathogen at different time points (28).

### Adhesion Assay

The Caco-2 cells and IPEC-J2 cells were cultivated as described by Dowdell et al. (29). Briefly, the cells were plated into 24-well plates. The bacterial pellets from an overnight culture were washed 3 times in PBS and then each pellet was finally resuspended to a concentration of 10^8^ CFU/ml in PBS with fluorescein isothiocyanate (FITC) (100 μg/ml) at 37°C for 1 h in dark. For removing unattached FITC, labeled bacteria were washed 4 times with PBS. The monolayers of cells were washed 3 times with PBS. About 10^6^ CFU/ml labeled bacteria were added to the cells and the fluorescence intensity was measured by a micro-volume spectrophotometer, with an optical absorption wavelength of 485 nm and an emission wavelength of 530 nm. The cells were cultured at 37°C with 5% CO_2_ for 1 h and then washed 3 times using PBS to remove unattached bacteria. Each well of 24-well tissue culture plates was added 0.1 ml of trypsin and the plates were incubated for 10 min to digest the cells completely. Finally, the cell culture medium with 20% FBS was added to stop the reaction and the fluorescence intensity was measured. The adhesion rate was calculated by the equation described as A/A0 × 100, where A0 represents the fluorescence intensity before adhesion and A is the fluorescence intensity after adhesion.

### Detection of Anti-pathogenic Activities

The antimicrobial activities of the LAB isolates against three kinds (six strains) of pathogenic bacteria were tested using the Oxford cup assay as described previously (30). Briefly, 15 ml of 1.5% (w/v) agar medium was poured onto a plate and solidified. Then 1% of each pathogen strain (10^7^ CFU/ml) was inoculated into 15 ml of 0.8% (w/v) of BHI agar at 45–50°C. The mixture was poured onto the agar medium and solidified. Four Oxford cups (6 mm) were put on the BHI agar surface and 100 μl of culture was poured into three, while the fourth was used as a control (100 μl of sterile water). The supernatant was diffused for 4 h at RT, followed incubating for 20 h at 37°C. The bacterial inhibition rings on plates were measured using a vernier caliper. The experiments were performed in triplicate.

### Safety Assessment of LAB

#### Hemolytic Activity Analysis

The LAB isolates were cultured in MRS medium for 18–24 h at 37°C. Streak plate methods were performed on sheep blood agar plates (Oxoid, Germany) to analyze hemolytic activity. And then the plates were incubated at 37°C. The appearance of clear zones around the bacteria colonies was indicated as α-, β-, or γ-hemolysis.

#### Detection of Potential Virulence Factors

Since *Enterococcus* emerge as the major opportunistic pathogen of humans and animals, the occurrence of virulence determinants was detected. The absence/presence of virulence factor genes for the isolated strains M2-3, M5-8, and M6-29 was performed using PCR amplification with primers and conditions reported previously (26, 31). The primers and reaction conditions of PCR for the virulence factors were listed in Table 1 (32–39).

**Table 1:** PCR primers and the annealing temperatures used to detect the putative virulence genes in the isolated LAB strains.

**Primers**	**Sequences (5′-3′)**	**Amplicon size (bp)**	**Tm (°C)**	**References**
Ace	F: CAGGCCAACATCAAGCAACA	125	65	(32)
	R: GCTTGCCTCGCCTTCTACAA			
Agg	F: AAGAAAAAGAAGTAGACCAAC	1,553	53	(33)
	R: AAACGGCAAGACAAGTAAATA			
Asa1	F: GCACGCTATTACGAACTATGA	375	56	(34)
	R: TAAGAAAGAACATCACCACGA			
AtpA	F: CCAGGTCGTGAAGCTTATCC	110	63	(35)
	R: GGTAAGGCCGTCATTGAACC			
Cfa 1	F: ACGACCTGTTGTTCGACCTG	150	63	(35)
	R: ACGACCTGTTGTTCGACCTG			
Cpd	F: TGGTGGGTTATTTTTCAATTC	782	50	(36)
	R: TACGGCTCTGGCTTACTA			
CylA	F: ACTCGGGGATTGATAGGC	688	60	(34)
	R: GCTGCTAAAGCTGCGCTT			
ClyB	F: ATTCCTACCTATGTTCTGTTA	843	56	(34)
	R: AATAAACTCTTCTTTTCCAAC			
Ebp	F: AATGTGTTAAACCATCAAGGGAAT	372	62	(37)
	R: ACTCCTTTTTGAACTTCACCAATC			
EspA	F: TTTGGGGCAACTGGAATAGT	407	60	(32)
	R: CCCAGCAAATAGTCCATCAT			
EfaAfs	F: GACAGACCCTCACGAATA	705	56	(36)
	R: AGTTCATCATGCTGTAGTA			
Fsr A	F: TGATGATGATTGATTGATGGAC	744	60	(38)
	R: ATTACAAGTGGCACACCAGGAC			
Fsr B	F: TGGACAAAGTATTATCTAACCG	729	57	(38)
	R: CACACCATCACTGACTTTTGC			
Fsr C	F: ATCGTGTGTTAGAAAATAGC	1,344	52	(38)
	R: ACGAATCACAACCACTAAGTC			
GelE	F: CGAAGTTGGAAAAGGAGGC	372	50	(32)
	R: GGTGAAGAAGTTACTCTGA			
GroEL	F: GTTTGATCGCGGCTATCTGA	150	55	(39)
	R: CCTTGTTGMACGATTTCTTG			
HisD	F: TGAACCACTCGGTGACTACG	150	62	(35)
	R: GGAGCTTCCTTAGCCAAAGC			
HyI	F: ACAGAAGAGCTGCAGGAAATG	276	62	(34)
	R: GACTGACGTCCAAGTTTCCAA			
MleS	F: ACAAGGTCTCAGCGTTCAGC	140	64	(35)
	R: GACTGGGATTCCAGCTGATG			
SprE	F: GGTAAACCAACCAAGTGAATC	300	57	(32)
	R:R: TTCTTCCGATTGACGCAAAA			

#### Screening of Antibiotic Resistance of LAB

The antibiotics employed in this study were erythromycin (15 μg), streptomycin (10 μg), cefotaxime (30 μg), kanamycin (30 μg), cefradine (30 μg), gentamicin (10 μg), ciprofloxacin (5 μg), amoxicillin (10 μg), chloramphenicol (30 μg), clindamycin (2 μg), ampicillin (10 μg), cotrimoxazole (25 μg), tetracycline (30 μg), ceftazidime (30 μg), and ceftriaxone (30 μg) (Oxoid, UK). The antibiotics susceptibility was tested for all the isolated LAB using Kirby-Bauer disk diffusion test (40). Plates were incubated at 37°C for 24 h and then the diameters of the inhibition zones were measured. The resistance of the LAB strains was interpreted using the guiding principles of the Clinical and Laboratory Standards Institute (CLSI 2014).

### *In vivo* Safety Evaluation

Five groups of 5 mice each were orally administered with isolates M5-8, M6-29, M4-7, M2-71, or M2-3 at a concentration of 10^9^ CFU/100 μl, and a control group of mice were fed with 100 μl PBS. After 21 days of continuous LAB supplementation, the parameters of general health status including body weight gain (BWG) and organ index were calculated to assess the safety of the LAB. The spleen, liver, and kidney of the mice were collected and determined for organ index as follows: weight of organ/ bodyweight of the mice (41).

### Statistical Analysis

The data analysis was performed using the program SPSS 22 Statistics. A one-way analysis of variance (ANOVA) was used to compare the data. Statistical significance was defined at *P* < 0.05.

## Results

### Isolation of LAB

In this study, 192 pure bacterial colonies were obtained from various GI compartments of wild boar. These colonies were isolated from the jejunum and duodenum (71 isolates), the ileum (2 isolates), the ileocecal apertures (23 isolates), the cecum (35 isolates), and the colon (61 isolates), respectively. To identify these selected isolates, 16S rDNA gene sequencing analysis was performed. The results revealed that there are 156 LAB isolates that belonged to five species, including *Lactobacillus mucosae, Lactobacillus salivarius, Enterococcus hirae, E. durans*, and *E. faecium*.

### Acid and Bile Salt Tolerance of LAB Isolates

The survivability in stomach and intestine is an important characteristic needed for probiotics. The acidic and bile salt conditions had different effects on the growth of all 156 selected isolates. Five isolates showed well-tolerance in both bile salt and low pH (Table 2). The sequences for the amplified 16S rDNA of five candidate strains were listed in Supplementary Material. Thus, we selected these five isolates for further investigation.

**Table 2:** The acid and bile salt tolerance of the LAB isolates of various origins and species.

**Isolates**	**Origin (Gut compartment)**	**Species**	**Survival rate (%) at pH 3.0**	**Survival rate (%) at 0.3% bile salt**
M2-3	Duodenum	*E. durans*	80.01	56.32
M2-71		*L. salivarius*	70.67	88.11
M4-7	Ileocecal aperture	*L. mucosae*	58.45	61.20
M5-8	Cecum	*E. hirae*	53.99	62.79
M6-29	Colon	*E. faecium*	55.93	67.88

### Autoaggregation and Coaggregation

**Figure 1:**
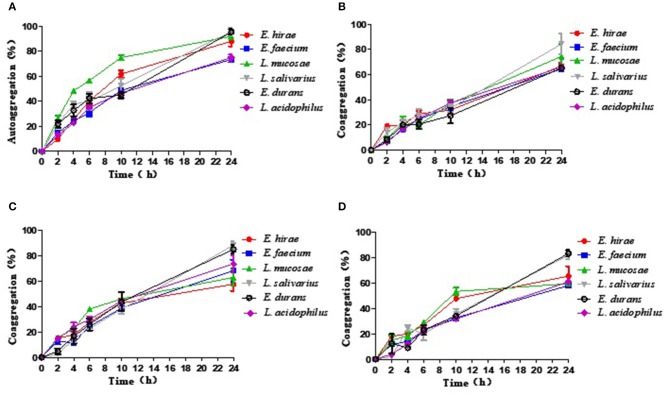
The percentage of autoaggregation **(A)** and coaggregation with enteropathogenic *Escherichia coli* **(B)**, *Salmonella typhimurium* **(C)**, or *Staphylococcus aureus* **(D)** by five lactic acid bacteria isolates. Data are represented as means ± standard deviations.

The results of autoaggregation and coaggregation detected for these five LAB were shown in Figure 1. The results indicated that each strain can autoaggregate and the percentage of autoaggregation increased over time. Among these probiotic strains, *L. salivarius* M2-71 showed the highest autoaggregation percentage of 95.6 ± 4.61% at 24 h (Figure 1A). Coaggregation analysis was tested using three different indicator strains at different time points. All the tested probiotic strains showed the capacities for aggregation with the tested pathogenic bacteria, but the coaggregation rates were confirmed to be strain-specific and time-dependent. Compared to the tested strains, M2-71 showed the maximum coaggregation percentages with enteropathogenic *E. coli* and *S. typhimurium* (Figures 1B,C), and *E. durans* M2-3 exhibited the highest coaggregation percentage with *S. aureus* (Figure 1D).

### Adhesion to Cells by LAB

**Figure 2:**
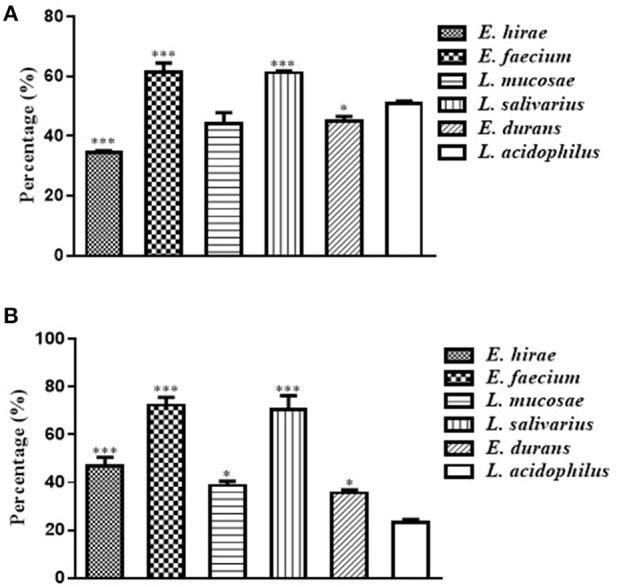
Adhesion abilities of selected lactic acid bacteria to Caco-2 cells **(A)** and IPEC-J2 cells **(B)**. Data are represented as means ± standard deviations. *P < 0.05; ***P < 0.001.

The adhesion ability to Caco-2 cells by these five selected isolates was shown in Figure 2A. The percentages of adhesion changed according to the isolate strains, ranging from 34.49 ± 0.81% to 61.49 ± 4.23%. Compared with the reference strain ATCC 4356, *L. salivarius* M2-71 and *E. faecium* M6-29 exhibited significantly higher adhesion rates (*P* < 0.001), while the adhesion rate of *E. hirae* M5-8 was relatively lower (*P* < 0.001) (Figure 2A). Figure 2B showed the adhesion capacity of the isolates to IPEC-J2 cells. The adhesion rates of M2-71 and M6-29 were significantly higher than that of reference strain (*P* < 0.001), while *E. durans* M2-3 exhibited relatively lower adhesion rate (*P* < 0.05).

### Antibacterial Activities of LAB

The strains were tested for their antimicrobial activities against different bacterial pathogens and the results were shown in Table 3. Among the five LAB isolates, *L. salivarius* M2-71 exhibited the strongest antimicrobial activity (inhibition zone diameters > 17 mm) toward all of the six indicator bacteria.

**Table 3:** The inhibitory effects of selected LAB strains against pathogenic microorganisms.

**Isolates**	**Indicator pathogens**					
	***E. coli***	***E. coli***	***S. typhimurium***	***S. typhimurium***	***S. aureus***	***S. aureus***
	**BNCC 337304**	**ATCC 8379**	**ATCC 14028**	**ATCC 19585**	**ATCC 6538P**	**ATCC 25923**
*E. durans*	14.19 ± 0.31	14.03 ± 0.09	14.67 ± 0.43	14.78 ± 0.28	13.76 ± 0.39	14.33 ± 0.66
*L. salivarius*	17.16 ± 0.21	18.93 ± 0.31	19.31 ± 0.28	18.87 ± 0.37	17.97 ± 0.15	17.05 ± 0.30
*L. mucosae*	16.11 ± 0.39	15.61 ± 0.11	14.30 ± 0.51	13.58 ± 0.19	14.79 ± 0.14	15.86 ± 0.28
*E. hirae*	12.88 ± 0.45	13.23 ± 0.29	14.73 ± 0.20	13.32 ± 0.46	13.24 ± 0.27	13.63 ± 0.08
*E. faecium*	13.35 ± 0.12	13.09 ± 0.32	14.77 ± 0.38	15.12 ± 0.31	13.68 ± 0.43	12.64 ± 0.26
*L. acidophilus*	14.99 ± 0.25	15.76 ± 0.34	14.37 ± 0.36	15.13 ± 0.34	12.77 ± 0.21	14.12 ± 0.20

*Values are means ± standard deviations of triplicates (mm)*.

### Safety Evaluation

All the isolates showed no hemolytic activity and none of the isolated *Enterococcus* strains harbored virulence factors genes (data not shown).

The antibiotic resistance of the LAB isolates against 15 tested antibiotics were shown in Table 4. In view of the results, all the five LAB exhibited the capacity to resist the impact of cotrimoxazole and tetracycline. On the other hand, they were all susceptible to chloramphenicol and amoxicillin. These strains were characterized by multiple resistances to at least three antibiotics. The *E. faecium* M6-29 strain was resistant to eleven antibiotics.

**Table 4:** Distribution of inhibition zone diameter range (mm) of LAB isolates using the disk diffusion testing of 15 antimicrobial agents.

**Isolates**	**Inhibition zone diameter (mm)**														
	**CTX**	**E**	**K**	**S**	**VI**	**AMX**	**C**	**CC**	**CIP**	**P**	**GM**	**SXT**	**TE**	**CAZ**	**CRO**
*E. durans*	I	S	R	R	I	S	S	R	S	S	R	R	R	S	I
*L. salivarius*	S	I	R	R	S	S	S	I	I	S	R	R	R	R	R
*L. mucosae*	R	R	R	R	R	S	S	R	I	S	R	R	R	R	R
*E. hirae*	R	I	I	S	I	S	S	S	I	S	R	I	R	I	I
*E. faecium*	I	R	R	R	R	S	S	R	I	R	R	R	R	R	R

Interpretation of the inhibition zone diameters are the resistance (R), susceptibility (S), and intermediate (I) according to CLSI (2014).

*Cefotaxime (CTX), erythromycin (E), kanamycin (K), streptomycin (S), cefradine (VI), amoxicillin (AMX), chloramphenicol (C), clindamycin (CC), ciprofloxacin (CIP), ampicillin (P), gentamicin (GM), cotrimoxazole (SXT), tetracycline (TE), ceftazidime (CAZ), and ceftriaxone (CRO)*.

**Figure 3:**
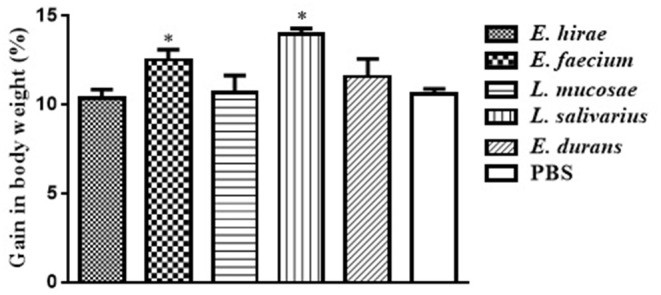
Effect of probiotic supplementation on body weight gain of experimental mice. Data are represented as means ± standard deviations. *P < 0.05.

**Figure 4:**
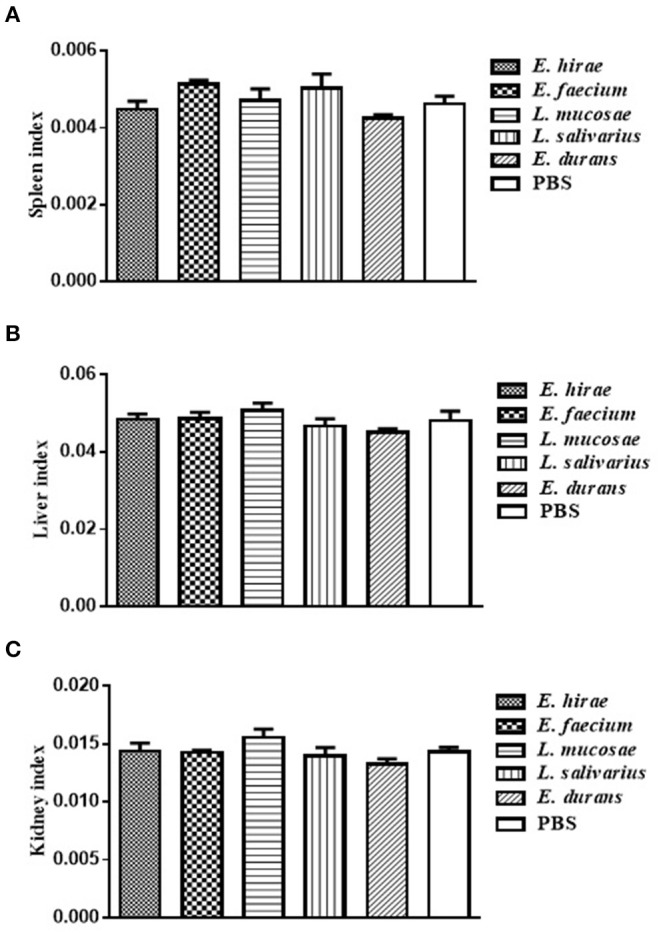
The indices analysis of spleen **(A)**, liver **(B)**, and kidney **(C)** in all the experimental groups. Data are represented as means ± standard deviations.

Compared with control group, no significant difference was observed in BWG of mice treated with various isolates on days 7 and 14 (data not shown). The percentage of BWG of the mice treated with *L. salivarius* M2-71 or *E. faecium* M6-29 was significantly higher (*P* < 0.05) than that of the control mice on day 21 (Figure 3). In addition, no significant difference in organ index among the different groups was found, including spleen (Figure 4A), liver (Figure 4B), or kidney (Figure 4C).

## Discussion

As an increasing consumer demand for livestock products, there has been a great interest in probiotics application for health promotion and performance improvement of livestock (31). Currently, LAB have been used as probiotics to treat intestinal diseases in humans and animals since they promote host intestinal health by developing a balance of intestinal microbes (42). And in recent years, researches have confirmed that the addition of probiotics to feed supplements is a promising approach for exerting a positive effect on the pigs in different growth stages (43). However, to ensure the efficiency and safety of these bacteria, they must be systematically identified and characterized. The purpose of our research was to analyze the potential probiotic characteristics and evaluate the safety of the LAB isolated from the wild boar in order to explore potentials for the development of feed additives for animals. This is the first report, to our best knowledge, to evaluate the probiotic characteristics of the LAB strains isolated from wild boar.

The bile salt in the duodenum and the acid condition of the stomach has been reported as the biggest obstacles to the survival of LAB in the GI tract of host (44). In the study, the different levels of resistance to bile salt of five isolates may depend on the expression of bile resistance-related proteins in the LAB cells (45). The isolates were also found to vary in the survivability in acidic conditions. These results are probably due to the species- and/or strain-dependent acid-tolerance mechanism (26) with certain bacterial proteins that might provide the resistance (46).

In this study, the five LAB strains screened by acid and bile salt resistance test belong to different species and correspond to those commonly found in pigs. *L. mucosae*, the well-known inhabitants of the GI of domestic pigs, are also frequently isolated from the digestive tract of wild boar (47). The *L. mucosae* LM1 strain isolated from pig feces has been considered as potential probiotic because of its adhesion to pig ileum mucous and inhibition of intestinal pathogens *in vitro* (28, 48). *Lactobacillus salivarius* as common gut inhabitants of raised pigs have been proven to offer benefits for gut health of pigs (49). The LAB isolated from wild boar in this study colonized in their host intestinal tract. Therefore, it is expected that these LAB isolates could be more adaptable to internal environment than other LAB. *Enterococcus* are also autochthonous flora in the GI tract of host and have different useful functions in dairy industry. Beneficial effects of the addition of *Enterococcus* to diets include improvement of the pig's intestinal microbiota and performance, and immune modulation (50).

Autoaggregation and coaggregation of probiotics play important roles in preventing pathogens from surface colonization (21). Autoaggregation can enable microorganisms which belong to the same species to join self-forming groups, and this phenomenon usually associated with microorganisms binding to intestinal mucosa (51). And coaggregation is the intercellular adhesion between different strains, which is also bound up with the ability of interacting with pathogens. Therefore, aggregation may constitute one kind of defense mechanisms of host for anti-infection (52). In this study, five LAB isolated from the same wild boar were observed to have different levels of autoaggregation and coaggregation. This may be a result of complex interplay among bacteria surface molecules, such as proteins, and secreted factors, etc. A previous study has confirmed that any potential aggregation phenotype depends on possible internal factors and the environment (53). The components related to the aggregation phenotype of these isolates need to be identified. Moreover, LAB expressing the aggregation-promoting factors could further promote eliminating pathogens via balancing gut microbial ecosystems and the coaggregation mechanism. Thus, the isolated LAB in this work possessing the ability to autoaggregate and coaggregate pathogens could be beneficial to the intestinal health. Likewise, the high coaggregation level can facilitate the presence of these strains in the intestinal tract of animals.

The ability to remain viable and adhere to the intestine of host is considered to be a key factor in many of the recommendations for the health benefits of probiotics (54). Adherence not only enables probiotics to live longer in the GI tract and enhances the interactions of bacteria and host, but also helps itself to overcome gastric motility (55). Therefore, the adhesion ability to mucosal surfaces and epithelial cells is a crucial feature of probiotics. A possible explanation to the adhesive ability of isolates varying from strain to strain in our study is that each of the strains has specific cell-surface molecules to play roles in the ability of adhesion. These molecules may mediate attachment to intestinal mucosa and regulate the immune system. Al Seraih et al. previously reported that high autoaggregation ability is correlated with strong adhesion (56). Similar results have been observed in the current study that the *L. mucosae* M6-29 and *L. salivarius* M2-71, with strong aggregation ability, have stronger capacity to adhere to cells than other isolates. It is supposed that aggregation factors which favorably increase H-bonding between and among cell surfaces have correlation with increased general adhesion ability (29). Our results support the hypothesis that a notable correlation was observed between aggregation and adhesion. Although the relation of the aggregation and adhesion was not characterized in this study, it would be an interesting topic for the next project.

A large amount of evidences suggest that certain probiotic strains can confer the ability of anti-infection with intestinal pathogenic microbes (57–59). As more and more concerns have been paid to the prevalence of antibiotic resistance, the antibacterial activity against pathogenic bacteria has attracted extensive attention (60). These strains as putative probiotics should have antimicrobial activities against both Gram-negative and Gram-positive pathogenic microorganisms. Among our LAB isolated from wild boar, *L. salivarius* M2-71 had the strongest antibacterial activity and a significant co-aggregation ability. Actually, antimicrobial activities are also related to coaggregation. The intimate contact between probiotics and pathogenic bacteria occurs during co-aggregation and the anti-microbial substances produced by the former can inhibit pathogens (61). In addition, the reports of the antiviral and anticancer activities are good examples of much broader than originally considered health-promoting activities attributed to probiotic LAB (62, 63). Although the antiviral and anticancer abilities of the isolated LAB were not characterized in this study, it would be an interesting topic for further studies.

Although LAB as food-grade microorganisms are safe to use with a long history, potential virulence-associated factors must be considered and assessed. In this research, none of the three *Enterococcus* isolates causes the lysis of erythrocytes of sheep blood or harbors virulence factors, and thus they can be regarded as the candidates for safe probiotics. The effects on the general health of animals are used to evaluate the safety profile of probiotic isolates. In order to assess the effects of probiotics on overall health condition, body weight gain and organ indices have been established and measured. No differences in organ indices, noticeable abnormal behavior, and loss in body weight were monitored after 3-week feeding with the LAB strains, indicating that these five isolates administered did not adversely affect the general health of mice. At the same time, M6-29 and M2-71 supplementation can increase growth performance to some extent. Our results further support the increasing evidence that LAB supplementation may have a healthy and beneficial effect on the animals.

Probiotics are known to contain mobile and intrinsic genetic factors that enable them to develop resistance to various antibiotics (64). The antibiotic resistance in these beneficial microbes is thought to be beneficial to the survival of antibiotics in the GI tract. The probiotics with endogenous resistance can restore intestinal flora after antibiotic treatment (65). The resistance of LAB against different antibiotics is one of the most important factors for safety assessment (66). Klose et al. described for the first time selective isolation of intrinsically vancomycin-resistant *Lactobacillus* species from the intestine of wild boar, and determined the prevalence of antibiotic resistance (47). The similar results were found in current study that the isolates were resistant to tetracycline, while susceptible to chloramphenicol. The most probable encounter route of domestic pigs with antibiotics will be through feed, water, and antibiotics used as prophylaxis. But it is not clear how wild boar acquire antibiotic-resistant LAB in their GI tracts.

## Conclusion

In this study, the LAB isolated from wild boar are safe and possess probiotic properties including the tolerance of bile salt and acid, autoaggregation and coaggregation capabilities, adherence abilities, and anti-pathogen activities. *Lactobacillus salivarius* M2-71 exhibited a strong aggregation and effective adherence to Caco-2 and IPEC-J2 cells. The results indicate that these five LAB strains, especially M2-71, have probiotic properties and the effects on animals as feed additives can be further study.

## Data Availability Statement

All datasets generated for this study are included in the article/Supplementary Material.

## Author Contributions

ML, YS, and H-JQ designed this study. ML and YW wrote the manuscript. ML, YW, HC, and YL performed the experiments. All authors reviewed this manuscript.

### Conflict of Interest

The authors declare that the research was conducted in the absence of any commercial or financial relationships that could be construed as a potential conflict of interest.

## References

[1] Mehdi Y Letourneau-Montminy MP Gaucher ML Chorfi Y Suresh G Rouissi T. Use of antibiotics in broiler production:Global impacts and alternatives. Anim Nutr. (2018) 4:170–8. 10.1016/j.aninu.2018.03.002 30140756 PMC6103476

[2] Al-Khalaifa H Al-Nasser A Al-Surayee T Al-Kandari S Al-Enzi N Al-Sharrah T. Effect of dietary probiotics and prebiotics on the performance of broiler chickens. Poult Sci. (2019) 98:4465–79. 10.3382/ps/pez282 31180128

[3] Wang A Ran C Wang Y Zhang Z Ding Q Yang Y. Use of probiotics in aquaculture of China-a review of the past decade. Fish Shellfish Immunol. (2019) 86:734–55. 10.1016/j.fsi.2018.12.026 30553887

[4] Kim DH Jeong D Kang IB Lim HW Cho Y Seo KH. Modulation of the intestinal microbiota of dogs by kefir as a functional dairy product. J Dairy Sci. (2019) 102:3903–11. 10.3168/jds.2018-15639 30827566

[5] Kim JA Bayo J Cha J Choi YJ Jung MY Kim DH. Investigating the probiotic characteristics of four microbial strains with potential application in feed industry. PLoS ONE. (2019) 14:e0218922. 10.1371/journal.pone.0218922 31242260 PMC6594638

[6] Landete JM. A review of food-grade vectors in lactic acid bacteria: from the laboratory to their application. Crit Rev Biotechnol. (2017) 37:296–308. 10.3109/07388551.2016.1144044 26918754

[7] Endo A Tanizawa Y Arita M. Isolation and identification of lactic acid bacteria from environmental samples. Methods Mol Biol. (2019) 1887:3–13. 10.1007/978-1-4939-8907-2_1 30506244

[8] Isolauri E Sutas Y Kankaanpaa P Arvilommi H Salminen S. Probiotics: effects on immunity. Am J Clin Nutr. (2001) 73:444S−50S. 10.1093/ajcn/73.2.444s 11157355

[9] Jager R Purpura M Farmer S Cash HA Keller D. Probiotic *bacillus coagulans* GBI-30, 6086 improves protein absorption and utilization. Probiot Antimicrob Proteins. (2018) 10:611–5. 10.1007/s12602-017-9354-y 29196920 PMC6208742

[10] Isaacs K Herfarth H. Role of probiotic therapy in IBD. Inflamm Bowel Dis. (2008) 14:1597–605. 10.1002/ibd.20465 18421762

[11] Miller LE Ouwehand AC Ibarra A. Effects of probiotic-containing products on stool frequency and intestinal transit in constipated adults: systematic review and meta-analysis of randomized controlled trials. Ann Gastroenterol. (2017) 30:629–39. 10.20524/aog.2017.0192 29118557 PMC5670282

[12] Alard J Peucelle V Boutillier D Breton J Kuylle S Pot B. New probiotic strains for inflammatory bowel disease management identified by combining *in vitro* and *in vivo* approaches. Benef Microbes. (2018) 9:317–31. 10.3920/BM2017.0097 29488412

[13] Lakritz JR Poutahidis T Levkovich T Varian BJ Ibrahim YM Chatzigiagkos A. Beneficial bacteria stimulate host immune cells to counteract dietary and genetic predisposition to mammary cancer in mice. Int J Cancer. (2014) 135:529–40. 10.1002/ijc.28702 24382758 PMC4131439

[14] Niibo M Shirouchi B Umegatani M Morita Y Ogawa A Sakai F. Probiotic *Lactobacillus gasseri* SBT2055 improves insulin secretion in a diabetic rat model. J Dairy Sci. (2019) 102:997–1006. 10.3168/jds.2018-15203 30471910

[15] Aswathy RG Ismail B John RP Nampoothiri KM. Evaluation of the probiotic characteristics of newly isolated lactic acid bacteria. Appl Biochem Biotechnol. (2008) 151:244–55. 10.1007/s12010-008-8183-6 18592412

[16] Sirichokchatchawan W Pupa P Praechansri P Am-In N Tanasupawat S Sonthayanon P. Autochthonous lactic acid bacteria isolated from pig faeces in Thailand show probiotic properties and antibacterial activity against enteric pathogenic bacteria. Microb Pathog. (2018) 119:208–15. 10.1016/j.micpath.2018.04.031 29678738

[17] Renaud DL Kelton DF Weese JS Noble C Duffield TF. Evaluation of a multispecies probiotic as a supportive treatment for diarrhea in dairy calves: a randomized clinical trial. J Dairy Sci. (2019) 102:4498–505. 10.3168/jds.2018-15793 30852016

[18] Parsa M Nosrati M Javandel F Seidavi A Khusro A Salem AZM The effects of dietary supplementation with different levels of Microzist as newly developed probiotics on growth performance, carcass characteristics, and immunological organs of broiler chicks. J Appl Anim Res. (2018) 46:1097–102. 10.1080/09712119.2018.1467835

[19] Laukova A Kandricakova A Scerbova J. Use of bacteriocin-producing, probiotic strain *Enterococcus faecium* AL41 to control intestinal microbiota in farm ostriches. Lett Appl Microbiol. (2015) 60:531–5. 10.1111/lam.12409 25732357

[20] Peng Y Shi Q Wang Y Zhang F Ji Z Zhang J. Dietary probiotics have different effects on the composition of fecal microbiota in farmed raccoon dog (Nyctereutes procyonoides) and silver fox (Vulpes vulpes fulva). BMC Microbiol. (2019) 19:109. 10.1186/s12866-019-1491-x 31126241 PMC6534910

[21] Sharma K Attri S Goel G. Selection and evaluation of probiotic and functional characteristics of autochthonous lactic acid bacteria isolated from fermented wheat flour dough *babroo*. Probiotics & Antimicro. Prot. (2019). 11:774–84. 10.1007/s12602-018-9466-z 30220016

[22] do Carmo MS Noronha FM Arruda MO Costa EP Bomfim MR Monteiro AS. *Lactobacillus fermentum* ATCC 23271 displays *in vitro* inhibitory activities against *Candida spp*. Front Microbiol. (2016) 7:1722. 10.3389/fmicb.2016.01722 27833605 PMC5082230

[23] Sarra PG Dellaglio F. Colonization of a human intestine by four different genotypes of *Lactobacillus acidophilus*. Microbiologica. (1984) 7:331–9. 6439978

[24] Bin Masalam MS Bahieldin A Alharbi MG Al-Masaudi S Al-Jaouni SK Harakeh SM. Isolation, molecular characterization and probiotic potential of lactic acid bacteria in saudi raw and fermented milk. Evid Based Complement Alternat Med. (2018) 2018:7970463. 10.1155/2018/7970463 30147735 PMC6083559

[25] Dowarah R Verma AK Agarwal N Singh P Singh BR. Selection and characterization of probiotic lactic acid bacteria and its impact on growth, nutrient digestibility, health and antioxidant status in weaned piglets. PLoS ONE. (2018) 13:e0192978. 10.1371/journal.pone.0192978 29518093 PMC5843174

[26] Nami Y Vaseghi Bakhshayesh R Mohammadzadeh Jalaly H Lotfi H Eslami S Hejazi MA. Probiotic properties of *Enterococcus* isolated from artisanal dairy products. Front Microbiol. (2019) 10:300. 10.3389/fmicb.2019.00300 30863379 PMC6400110

[27] Collado MC Grzeskowiak L Salminen S. Probiotic strains and their combination inhibit *in vitro* adhesion of pathogens to pig intestinal mucosa. Curr Microbiol. (2007) 55:260–5. 10.1007/s00284-007-0144-8 17657533

[28] Valeriano VD Parungao-Balolong MM Kang DK. *In vitro* evaluation of the mucin-adhesion ability and probiotic potential of *Lactobacillus mucosae* LM1. J Appl Microbiol. (2014) 117:485–97. 10.1111/jam.12539 24807045

[29] Dowdell P Chankhamhaengdecha S Panbangred W Janvilisri T Aroonnual A. Probiotic activity of *Enterococcus faecium* and *Lactococcus lactis* isolated from Thai fermented sausages and their protective effect against *Clostridium difficile*. Probiotics Antimicrob Proteins. (2019). [Epub ahead of print]. 10.1007/s12602-019-09536-7 30888623 PMC7306037

[30] Muhammad Z Ramzan R Abdelazez A Amjad A Afzaal M Zhang S. Assessment of the antimicrobial potentiality and functionality of *Lactobacillus plantarum* strains isolated from the conventional Inner Mongolian fermented cheese against foodborne pathogens. Pathogens. (2019) 8:E71. 10.3390/pathogens8020071 31117307 PMC6631976

[31] Aristimuño Ficoseco C Mansilla FI Maldonado NC Miranda H Fátima Nader-Macias ME Vignolo GM. Safety and growth optimization of lactic acid bacteria isolated from feedlot cattle for probiotic formula design. Front Microbiol. (2018) 9:2220. 10.3389/fmicb.2018.02220 30323790 PMC6172481

[32] Al-Talib H Zuraina N Kamarudin B Yean CY. Genotypic variations of virulent genes in *Enterococcus faecium* and *Enterococcus faecalis* isolated from three hospitals in Malaysia. Adv Clin Exp Med. (2015) 24:121–7. 10.17219/acem/38162 25923096

[33] Espeche MC Pellegrino M Frola I Larriestra A Bogni C Nader-Macías MEF. Lactic acid bacteria from raw milk as potentially beneficial strains to prevent bovine mastitis. Anaerobe. (2012) 18:103–9. 10.1016/j.anaerobe.2012.01.002 22261519

[34] Vankerckhoven V Van Autgaerden T Vael C Lammens C Chapelle S Rossi R. Development of a multiplex PCR for the detection of *asa1, gelE*, *cylA, esp*, and *hyl* genes in *Enterococci* and survey for virulence determinants among European hospital isolates of *Enterococcus faecium*. J Clin Microbiol. (2004) 42:4473–9. 10.1128/JCM.42.10.4473-4479.2004 15472296 PMC522368

[35] Šeme H Gjurači'c K Kos B Fujs Š Štempelj M Petkovi'c H. Acid resistance and response to pH-induced stress in two *Lactobacillus plantarum* strains with probiotic potential. Benef Microbes. (2015) 6:369–79. 10.3920/BM2014.0069 25380802

[36] Eaton TJ Gasson MJ. Molecular screening of *Enterococcus* virulence determinants and potential for genetic exchange between food and medical isolates. Appl Environ Microbiol. (2001) 67:1628–35. 10.1128/AEM.67.4.1628-1635.2001 11282615 PMC92779

[37] Sillanpää J Chang C Singh KV Montealegre MC Nallapareddy SR Harvey BR. Contribution of individual Ebp pilus subunits of *Enterococcus faecalis* OG1RF to pilus biogenesis, biofilm formation and urinary tract infection. PLoS ONE. (2013) 8:e68813. 10.1371/journal.pone.0068813 23874774 PMC3708956

[38] Qin Y Luo ZQ Smyth AJ Gao P Beck von Bodman S Farrand SK. Quorum-sensing signal binding results in dimerization of TraR and its release from membranes into the cytoplasm. EMBO J. (2000) 19:5212–21. 10.1093/emboj/19.19.5212 11013223 PMC302097

[39] Koirala R Taverniti V Balzaretti S Ricci G Fortina MG Guglielmetti S. Melting curve analysis of a groEL PCR fragment for the rapid genotyping of strains belonging to the *Lactobacillus casei* group of species. Microbiol Res. (2015) 173:50–8. 10.1016/j.micres.2015.01.001 25801971

[40] Adetoye A Pinloche E Adeniyi BA Ayeni FA. Characterization and anti-salmonella activities of lactic acid bacteria isolated from cattle faeces. BMC Microbiol. (2018) 18:96. 10.1186/s12866-018-1248-y 30165820 PMC6118008

[41] Li A Wang Y Li Z Qamar H Mehmood K Zhang L. Probiotics isolated from yaks improves the growth performance, antioxidant activity, and cytokines related to immunity and inflammation in mice. Microb Cell Fact. (2019) 18:112. 10.1186/s12934-019-1161-6 31217027 PMC6585042

[42] Nami Y Haghshenas B Haghshenas M Yari Khosroushahi A. Antimicrobial activity and the presence of virulence factors and bacteriocin structural genes in *Enterococcus faecium* CM33 isolated from ewe colostrum. Front Microbiol. (2015) 6:782. 10.3389/fmicb.2015.00782 26284059 PMC4518196

[43] Wang W Ganzle M. Toward rational selection criteria for selection of probiotics in pigs. Adv Appl Microbiol. (2019) 107:83–112. 10.1016/bs.aambs.2019.03.003 31128749

[44] Hsu TC Yi PJ Lee TY Liu JR. Probiotic characteristics and zearalenone-removal ability of a *Bacillus licheniformis* strain. PLoS ONE. (2018) 13:e0194866. 10.1371/journal.pone.0194866 29641608 PMC5895015

[45] Hamon E Horvatovich P Izquierdo E Bringel F Marchioni E Aoude-Werner D. Comparative proteomic analysis of *Lactobacillus plantarum* for the identification of key proteins in bile tolerance. BMC Microbiol. (2011) 11:63. 10.1186/1471-2180-11-63 21447177 PMC3073879

[46] Yu Z Zhang X Li S Li C Li D Yang Z. Evaluation of probiotic properties of *Lactobacillus plantarum* strains isolated from Chinese sauerkraut. World J Microbiol Biotechnol. (2013) 29:489–98. 10.1007/s11274-012-1202-3 23117677

[47] Klose V Bayer K Kern C Goelss F Fibi S Wegl G. Antibiotic resistances of intestinal *lactobacilli* isolated from wild boars. Vet Microbiol. (2014) 168:240–4. 10.1016/j.vetmic.2013.11.014 24326231

[48] Valeriano VD Bagon BB Balolong MP Kang DK. Carbohydrate-binding specificities of potential probiotic *Lactobacillus* strains in porcine jejunal (IPEC-J2) cells and porcine mucin. J Microbiol. (2016) 54:510–9. 10.1007/s12275-016-6168-7 27350617

[49] Sayan H Assavacheep P Angkanaporn K Assavacheep A. Effect of *Lactobacillus salivarius* on growth performance, diarrhea incidence, fecal bacterial population and intestinal morphology of suckling pigs challenged with F4^+^ enterotoxigenic *Escherichia coli*. Asian-Australas J Anim Sci. (2018) 31:1308–14. 10.5713/ajas.17.0746 29642683 PMC6043459

[50] Scharek L Guth J Reiter K Weyrauch KD Taras D Schwerk P. Influence of a probiotic *Enterococcus faecium* strain on development of the immune system of sows and piglets. Vet Immunol Immunopathol. (2005) 105:151–61. 10.1016/j.vetimm.2004.12.022 15797484

[51] Lukic J Strahinic I Milenkovic M Nikolic M Tolinacki M Kojic M. Aggregation factor as an inhibitor of bacterial binding to gut mucosa. Microb Ecol. (2014) 68:633–44. 10.1007/s00248-014-0426-1 24823989

[52] Zhang W Liu M Dai X. Biological characteristics and probiotic effect of *Leuconostoc lactis* strain isolated from the intestine of black porgy fish. Braz J Microbiol. (2013) 44:685–91. 10.1590/S1517-83822013005000053 24516418 PMC3910175

[53] Goh YJ Klaenhammer TR. Functional roles of aggregation-promoting-like factor in stress tolerance and adherence of *Lactobacillus acidophilus* NCFM. Appl Environ Microbiol. (2010) 76:5005–12. 10.1128/AEM.00030-10 20562289 PMC2916482

[54] Iniguez-Palomares C Jimenez-Flores R Vazquez-Moreno L Ramos-Clamont-Montfort G Acedo-Felix E. Protein-carbohydrate interactions between *Lactobacillus salivarius* and pig mucins. J Anim Sci. (2011) 89:3125–31. 10.2527/jas.2010-2996 21622872

[55] Jose NM Bunt CR Hussain MA. Comparison of microbiological and probiotic characteristics of *Lactobacilli* isolates from dairy food products and animal rumen contents. Microorganisms. (2015) 3:198–212. 10.3390/microorganisms3020198 27682086 PMC5023236

[56] Al Seraih A Belguesmia Y Cudennec B Baah J Drider D. *In silico* and experimental data claiming safety aspects and beneficial attributes of the bacteriocinogenic strain *Enterococcus faecalis* B3A-B3B. Probiotics Antimicrob Proteins. (2018) 10:456–65. 10.1007/s12602-017-9357-8 29168155

[57] Arques JL Rodriguez E Langa S Landete JM Medina M. Antimicrobial activity of lactic acid bacteria in dairy products and gut: effect on pathogens. Biomed Res Int. (2015) 2015:584183. 10.1155/2015/584183 25861634 PMC4378328

[58] Campana R van Hemert S Baffone W. Strain-specific probiotic properties of lactic acid bacteria and their interference with human intestinal pathogens invasion. Gut Pathog. (2017) 9:12. 10.1186/s13099-017-0162-4 28286570 PMC5338089

[59] Kumar R Seo BJ Mun MR Kim CJ Lee I Kim H. Putative probiotic *Lactobacillus spp*. from porcine gastrointestinal tract inhibit transmissible gastroenteritis coronavirus and enteric bacterial pathogens. Trop Anim Health Prod. (2010) 42:1855–60. 10.1007/s11250-010-9648-5 20623187 PMC7089342

[60] Lee JS Chung MJ Seo JG. *In vitro* evaluation of antimicrobial activity of lactic acid bacteria against *Clostridium difficile*. Toxicol Res. (2013) 29:99–106. 10.5487/TR.2013.29.2.099 24278635 PMC3834449

[61] Arena MP Capozzi V Russo P Drider D Spano G Fiocco D. Immunobiosis and probiosis:antimicrobial activity of lactic acid bacteria with a focus on their antiviral and antifungal properties. Appl Microbiol Biotechnol. (2018) 102:9949–58. 10.1007/s00253-018-9403-9 30280241

[62] Drider D Bendali F Naghmouchi K Chikindas ML. Bacteriocins: not only antibacterial agents. Probiot Antimicrob Proteins. (2016) 8:177–82. 10.1007/s12602-016-9223-0 27481236

[63] Kaur M Singh H Jangra M Kaur L Jaswal P Dureja C. Lactic acid bacteria isolated from yak milk show probiotic potential. Appl Microbiol Biotechnol. (2017) 101:7635–52. 10.1007/s00253-017-8473-4 28879447

[64] Zheng M Zhang R Tian X Zhou X Pan X Wong A. Assessing the risk of probiotic dietary supplements in the context of antibiotic resistance. Front Microbiol. (2017) 8:908. 10.3389/fmicb.2017.00908 28579981 PMC5437161

[65] Zommiti M Connil N Hamida JB Ferchichi M. Probiotic characteristics of *Lactobacillus curvatus* DN317, a strain isolated from chicken ceca. Probiot Antimicrob Proteins. (2017) 9:415–24. 10.1007/s12602-017-9301-y 28741151

[66] Guo L Li T Tang Y Yang L Huo G. Probiotic properties of *Enterococcus* strains isolated from traditional naturally fermented cream in China. Microb Biotechnol. (2016) 9:737–45. 10.1111/1751-7915.12306 26200795 PMC5072190

