# Supplementary material for: Characterization of Lactic Acid Bacteria Isolated From the Gastrointestinal Tract of a Wild Boar as Potential Probiotics
Source: Front Vet Sci. 2020 Feb 11;7:49. doi: 10.3389/fvets.2020.00049 (PMC7026679; doi:10.3389/fvets.2020.00049)
Supplement: Supplementary file 1 [file Table_1.DOCX]

**16S rDNA gene sequences of five LAB isolates**

**16S rDNA gene sequence of *Enterococcus durans* M2-3**

ACTTCCCCCAATCATCTATCCCACCTTAGGCGGCTGGCTCCAAAAGGTTACCTCACCGACTTCGGGTGTTACAAACTCTCGTGGTGTGACGGGCGGTGTGTACAAGGCCCGGGAACGTATTCACCGCGGCGTGCTGATCCGCGATTACTAGCGATTCCGGCTTCATGTAGGCGAGTTGCAGCCTACAATCCGAACTGAGAGAAGCTTTAAGAGATTAGCTTAGCCTCGCGACTTCGCGACTCGTTGTACTTCCCATTGTAGCACGTGTGTAGCCCAGGTCATAAGGGGCATGATGATTTGACGTCATCCCCACCTTCCTCCGGTTTGTCACCGGCAGTCTTGCTAGAGTGCCCAACTAAATGATGGCAACTAACAATAAGGGTTGCGCTCGTTGCGGGACTTAACCCAACATCTCACGACACGAGCTGACGACAACCATGCACCACCTGTCACTTTGCCCCCGAAGGGGAAGCTCTATCTCTAGAGTGGTCAAAGGATGTCAAGACCTGGTAAGGTTCTTCGCGTTGCTTCGAATTAAACCACATGCTCCACCGCTTGTGCGGGCCCCCGTCAATTCCTTTGAGTTTCAACCTTGCGGTCGTACTCCCCAGGCGGAGTGCTTAATGCGTTAGCTGCAGCACTGAAGGGCGGAAACCCTCCAACACTTAGCACTCATCGTTTACGGCGTGGACTACCAGGGTATCTAATCCTGTTTGCTCCCCACGCTTTCGAGCCTCAGCGTCAGTTACAGACCAGAGAGCCGCCTTCGCCACTGGTGTTCCTCCATATATCTACGCATTTCACCGCTACACATGGAATTCCACTCTCCTCTTCTGCACTCAAGTCTCCCAGTTTCCAATGACCCTCCCCGGTTGAGCCGGGGGCTTTCACATCAGACTTAAGAAACCGCCTGCGCTCGCTTTACGCCCAATAAATCCGGACAACGCTTGCCACCTACGTATTACCGCGGCTGCTGGCACGTAGTTAGCCGTGGCTTTCTGGTTAGATACCGTCAAGGGATGAACAGTTACTCTCATCCTTGTTCTTCTCTAACAACAGAGTTTTACGATCCGAAAACCTTCTTCACTCACGCGGCGTTGCTCGGTCAGACTTTCGTCCATTGCCGAAGATTCCCTACTGCTGCCTCCCGTAGGAGTTTGGGCCGTGTCTCAGTCCCAATGTGGCCGATCACCCTCTCAGGTCGGCTATGCATCGTGGCCTTGGTGAGCCGTTACCTCACCAACTAGCTAATGCACCGCGGGTCCATCCATCAGCGGCACCGTAAAGCGCCTTTCAAATCAAAACCATGCGGTTTCGATTGTTATACGGTATTAGCACCTGTTTCCAAGTGTTATCCCCTTCTGATGGGCAGGTTACCCACGTGTTACTCACCCGTTCGCCACTCCTCTTTTTCCGGTGGAGCAAGCT

**16S rDNA gene sequence of *Lactobacillus salivarius* M2-71**

GGGGCTGGCGGGTGCTATACATGCAAGTCGAACGAAACTTTCTTACACCGAATGCTTGCATTCACCGTAAGAAGTTGAGTGGCGGACGGGTGAGTAACACGTGGGTAACCTGCCTAAAAGAAGGGGATAACACTTGGAAACAGGTGCTAATACCGTATATCTCTAAGGATCGCATGATCCTTAGATGAAAGATGGTTCTGCTATCGCTTTTAGATGGACCCGCGGCGTATTAACTAGTTGGTGGGGTAACGGCCTACCAAGGTGATGATACGTAGCCGAACTGAGAGGTTGATCGGCCACATTGGGACTGAGACACGGCCCAAACTCCTACGGGAGGCAGCAGTAGGGAATCTTCCACAATGGACGCAAGTCTGATGGAGCAACGCCGCGTGAGTGAAGAAGGTCTTCGGATCGTAAAACTCTGTTGTTAGAGAAGAACACGAGTGAGAGTAACTGTTCATTCGATGACGGTATCTAACCAGCAAGTCACGGCTAACTACGTGCCAGCAGCCGCGGTAATACGTAGGTGGCAAGCGTTGTCCGGATTTATTGGGCGTAAAGGGAACGCAGGCGGTCTTTTAAGTCTGATGTGAAAGCCTTCGGCTTAACCGGAGTAGTGCATTGGAAACTGGAAGACTTGAGTGCAGAAGAGGAGAGTGGAACTCCATGTGTAGCGGTGAAATGCGTAGATATATGGAAGAACACCAGTGGCGAAAGCGGCTCTCTGGTCTGTAACTGACGCTGAGGTTCGAAAGCGTGGGTAGCAAACAGGATTAGATACCCTGGTAGTCCACGCCGTAAACGATGAATGCTAGGTGTTGGAGGGTTTCCGCCCTTCAGTGCCGCAGCTAACGCAATAAGCATTCCGCCTGGGGAGTACGACCGCAAGGTTGAAACTCAAAGGAATTGACGGGGGCCCGCACAAGCGGTGGAGCATGTGGTTTAATTCGAAGCAACGCGAAGAACCTTACCAGGTCTTGACATCCTTTGACCACCTAAGAGATTAGGCTTTCCCTTCGGGGACAAAGTGACAGGTGGTGCATGGCTGTCGTCAGCTCGTGTCGTGAGATGTTGGGTTAAGTCCCGCAACGAGCGCAACCCTTGTTGTCAGTTGCCAGCATTAAGTTGGGCACTCTGGCGAGACTGCCGGTGACAAACCGGAGGAAGGTGGGGACGACGTCAAGTCATCATGCCCCTTATGACCTGGGCTACACACGTGCTACAATGGACGGTACAACGAGTCGCGAGACCGCGAGGTTTAGCTAATCTCTTAAAGCCGTTCTCAGTTCGGATTGTAGGCTGCAACTCGCCTACATGAAGTCGGAATCGCTAGTAATCGCGAATCAGCATGTCGCGGTGAATACGTTCCCGGGCCTTGTACACACCGCCCGTCACACCATGAGAGTTTGTAACACCCAAAGCCGGTGGGGTAACCGCAAGGAGCCAGCCGTCTAAGGTGGGACAGATGATTGGGGTGAAGTCGTAACAAGATGCCATCC

**16S rDNA gene sequence of *Lactobacillus mucosae* M4-7**

CGCAGGCACTTGTACGACTTCACCCTAATCATCTGCCCCACCTTAGGCGGCTAGCTCCCCGAAGGGTTACCCCACCGACTTTGGGTGTTGCAAACTCTCATGGTGTGACGGGCGGTGTGTACAAGGCCCGGGAACGTATTCACCGCGGCATGCTGATCCGCGATTACTAGCGATTCCGACTTCGTGCAGGCGAGTTGCAGCCTGCAGTCCGAACTGAGAACGGTTTTAAGAGATTAGCTTGCCCTCGCGAGTTCGCGACTCGTTGTACCGTCCATTGTAGCACGTGTGTAGCCCAGGTCATAAGGGGCATGATGATCTGACGTCGTCCCCACCTTCCTCCGGTTTGTCACCGGCAGTCTCACTAGAGTGCCCAACTGAATGCTGGCAACTAGTAACAAGGGTTGCGCTCGTTGCGGGACTTAACCCAACATCTCACGACACGAGCTGACGACGACCATGCACCACCTGTCATTGCGTTCCCGAAGGAAACGCCCTATCTCTAGGGTTGGCGCAAGATGTCAAGACCTGGTAAGGTTCTTCGCGTAGCTTCGAATTAAACCACATGCTCCACCGCTTGTGCGGGCCCCCGTCAATTCCTTTGAGTTTCAACCTTGCGGTCGTACTCCCCAGGCGGAGTGCTTAATGCGTTAGCTGCGGCACTGAAGGGCGGAAACCCTCCAACACCTAGCACTCATCGTTTACGGCATGGACTACCAGGGTATCTAATCCTGTTCGCTACCCATGCTTTCGAGCCTCAGCGTCAGTTGCAGACCAGACAGCCGCCTTCGCCACTGGTGTTCTTCCATATATCTACGCATTCCACCGCTACACATGGAGTTCCACTGTCCTCTTCTGCACTCAAGTCTGACAGTTTCCGATGCACTTCTTTGGTTAAGCCAAAGGCTTTCACATCAGACTTATCAAACCGCCTGCGCTCGCTTTACGCCCAATAAATCCGGATAACGCTTGCCACCTACGTATTACCGCGGCTGCTGGCACGTAGTTAGCCGTGACTTTCTGGTTAGATACCGTCACTGCGTGAACAGTTGCTCTCACGCACGTTCTTCTCTAACAACAGAGCTTTACGAGCCGAAACCCTTCTTCACTCACGCGGTGTTGCTCCATCAGGCTTGCGCCCATTGTGGAAGATTCCCTACTGCTGCCTCCCGTAGGAGTATGGACCGTGTCTCAGTTCCATTGTGGCCGATCAGTCTCTCAACTCGGCTACGCATCACAGCCTTGGTAGGCCGTTACCCTACCAACAAGCTAATGCGCCGCAGGTCCATCCCAAAGTGATAGCCGAAGCCATCTTTTAAATTTGAATCATGCGATTCAAATTGTTATGCGGTATTAGCATCTGTTTCCAAATGTTATCCCCCGCTTTGGGGCAGGTTACCTACGTGTTACTCACCCGTCCGCCACTCGCTGGTAAACCAACGTCAAGTCCGTGCAAGCACGTTCAATCAGTTGGGCCAACGCGTCGACTGCAGTATAGCACATGCCGCCG

**16S rDNA gene sequence of *Enterococcus hirae* M5-8**

TGCTCTTTGTTCGACTTCCCCCAATCATCTATCCCACCTTAGGCGGCTGGCTCCAAAAGGTTACCTCACCGACTTCGGGTGTTACAAACTCTCGTGGTGTGACGGGCGGTGTGTACAAGGCCCGGGAACGTATTCACCGCGGCGTGCTGATCCGCGATTACTAGCGATTCCGGCTTCATGTAGGCGAGTTGCAGCCTACAATCCGAACTGAGAGAAGCTTTAAGAGATTAGCTTAGCCTCGCGACTTTGCGACTCGTTGTACTTCCCATTGTAGCACGTGTGTAGCCCAGGTCATAAGGGGCATGATGATTTGACGTCATCCCCACCTTCCTCCGGTTTGTCACCGGCAGTCTTGCTAGAGTGCCCAACTAAATGATGGCAACTAACAATAAGGGTTGCGCTCGTTGCGGGACTTAACCCAACATCTCACGACACGAGCTGACGACAACCATGCACCACCTGTCACTTTGCCCCCGAAGGGGAAGCTCTATCTCTAGAGTGGTCAAAGGATGTCAAGACCTGGTAAGGTTCTTCGCGTTGCTTCGAATTAAACCACATGCTCCACCGCTTGTGCGGGCCCCCGTCAATTCCTTTGAGTTTCAACCTTGCGGTCGTACTCCCCAGGCGGAGTGCTTAATGCGTTAGCTGCAGCACTGAAGGGCGGAAACCCTCCAACACTTAGCACTCATCGTTTACGGCGTGGACTACCAGGGTATCTAATCCTGTTTGCTCCCCACGCTTTCGAGCCTCAGCGTCAGTTACAGACCAGAGAGCCGCCTTCGGCCACTGGTGTTCCTCCATATATCTACGCATTTCACCGCTACACATGGAATTCCACTCTCCTCTTCTGCACTCAAGTCTCCCAGTTTCCAATGACCCTCCCCGGTTGAGCCGGGGGCTTTCACATCAGACTTAAGAAACCGCCTGCGCTCGCTTTACGCCCAATAAATCCGGACAACGCTTGCCACCTACGTATTACCGCGGCTGCTGGCACGTAGTTAGCCGTGGCTTTCTGGTTAGATACCGTCAAGGGATGAACAGTTACTCTCATCCTTGTTCTTCTCTAACAACAGAGTTTTACGATCCGAAAACCTTCTTCACTCACGCGGCGTTGCTCGGTCAGACTTTCGTCCATTGCCGAAGATTCCCTACTGCTGCCTCCCGTAGGAGTTTGGGCCGTGTCTCAGTCCCAATGTGGCCGATCACCCTCTCAGGTCGGCTATGCATCGTCGCCTTGGTGAGCCGTTACCTCACCAACTAGCTAATGCACCGCGGGTCCATCCATCAGCGACACCCGAAAGCGCCTTTCAAATCAAAACCATGCGGTTTCGATTGTTATACGGTATTAGCACCTGTTTCCAAGTGTTATCCCCTTCTGATGGGCAGGTTACCCACGTGTTACTCACCCGTTCGCCACTCCTCTTTTTCCGGTGGAGCAAGCTCCGGTGGAAAAAGAAGCGTTCGACTTGCATGTATTAGGCACGCCGCACTCGCGCCCTATGAAGAAAGAAAAAAAAAAAAAAATAAAA

**16S rDNA gene sequence of *Enterococcus faecium* M6-29**

ATGACGACGTGCTATAATGCAGTCGAACGCTTCTTTTTCCACCGGAGCTTGCTCCACCGGAAAAAGAAGAGTGGCGAACGGGTGAGTAACACGTGGGTAACCTGCCCATCAGAAGGGGATAACACTTGGAAACAGGTGCTAATACCGTATAACAATCGAAACCGCATGGTTTTGATTTGAAAGGCGCTTTCGGGTGTCGCTGATGGATGGACCCGCGGTGCATTAGCTAGTTGGTGAGGTAACGGCTCACCAAGGCCACGATGCATAGCCGACCTGAGAGGGTGATCGGCCACATTGGGACTGAGACACGGCCCAAACTCCTACGGGAGGCAGCAGTAGGGAATCTTCGGCAATGGACGAAAGTCTGACCGAGCAACGCCGCGTGAGTGAAGAAGGTTTTCGGATCGTAAAACTCTGTTGTTAGAGAAGAACAAGGATGAGAGTAACTGTTCATCCCTTGACGGTATCTAACCAGAAAGCCACGGCTAACTACGTGCCAGCAGCCGCGGTAATACGTAGGTGGCAAGCGTTGTCCGGATTTATTGGGCGTAAAGCGAGCGCAGGCGGTTTCTTAAGTCTGATGTGAAAGCCCCCGGCTCAACCGGGGAGGGTCATTGGAAACTGGGAGACTTGAGTGCAGAAGAGGAGAGTGGAATTCCATGTGTAGCGGTGAAATGCGTAGATATATGGAGGAACACCAGTGGCGAAGGCGGCTCTCTGGTCTGTAACTGACGCTGAGGCTCGAAAGCGTGGGGAGCAAACAGGATTAGATACCCTGGTAGTCCACGCCGTAAACGATGAGTGCTAAGTGGTTGGAGGGTTTCCGCCCTTCAGTGCTGCAGCTAACGCATTAAGCACTCCGCCTGGGGAGTACGACCGCAAGGTTGAAACTCAAAGGAATTGACGGGGGCCCGCACAAGCGGTGGAGCATGTGGTTTAATTCGAAGCAACGCGAAGAACCTTACCAGGTCTTGACATCCTTTGACCACTCTAGAGATAGAGCTTCCCCTTCGGGGGCAAAGTGACAGGTGGTGCATGGTTGTCGTCAGCTCGTGTCGTGAGATGTTGGGTTAAGTCCCGCAACGAGCGCAACCCTTATTGTTAGTTGCCATCATTCAGTTGGGCACTCTAGCAAGACTGCCGGTGACAAACCGGAGGAAGGTGGGGATGACGTCAAATCATCATGCCCCTTATGACCTGGGCTACACACGTGCTACAATGGGAAGTACAACGAGTCGCGAAGTCGCGAGGCTAAGCTAATCTCTTAAAGCTTCTCTCAGTTCGGATTGCAGGCTGCAACTCGCCTGCATGAAGCCGGAATCGCTAGTAATCGCGGATCAGCACGCCGCGGTGAATACGTTCCCGGGCCTTGTACACACCGCCCGTCACACCACGAGAGTTTGTAACACCCGAAGTCGGTGAGGTAACCTTTTTGGAACCAGCCGCCTAAGGGGGAAAAAATGATTGGGGTGAAAT
